# Characterization of Immune Cell Subsets of Tumor Infiltrating Lymphocytes in Brain Metastases

**DOI:** 10.3390/biology10050425

**Published:** 2021-05-11

**Authors:** Priyakshi Kalita-de Croft, Haarika Chittoory, Tam H. Nguyen, Jodi M. Saunus, Woo Gyeong Kim, Amy E. McCart Reed, Malcolm Lim, Xavier M. De Luca, Kaltin Ferguson, Colleen Niland, Roberta Mazzieri, Riccardo Dolcetti, Peter T. Simpson, Sunil R. Lakhani

**Affiliations:** 1 UQ Centre for Clinical Research, Faculty of Medicine, University of Queensland, Herston, QLD 4029, Australia; h.chittoory@uq.edu.au (H.C.); j.saunus@uq.edu.au (J.M.S.); systaz02@gmail.com (W.G.K.); amy.reed@uq.edu.au (A.E.M.R.); m.lim@uq.edu.au (M.L.); m.deluca@uq.edu.au (X.M.D.L.); k.ferguson2@uq.edu.au (K.F.); c.niland@uq.edu.au (C.N.); p.simpson@uq.edu.au (P.T.S.); 2 QIMR Berghofer Medical Research Institute, Herston, QLD 4029, Australia; tamhong.nguyen@qimrberghofer.edu.au; 3 Diamantina Institute, Translational, Research Institute, The University of Queensland Diamantina Institute, Brisbane, QLD 4102, Australia; r.mazzieri@uq.edu.au (R.M.); Riccardo.Dolcetti@petermac.org (R.D.); 4 Peter MacCallum Cancer Centre, Melbourne, VIC 3000, Australia; 5 Sir Peter MacCallum Department of Oncology, The University of Melbourne, Parkville, VIC 3010, Australia; 6 Department of Microbiology and Immunology, The University of Melbourne, Parkville, VIC 3010, Australia; 7 Pathology Queensland, The Royal Brisbane and Women’s Hospital Herston, Herston, QLD 4029, Australia

**Keywords:** brain metastasis, immune checkpoint, tumor microenvironment, T-cells, TILs

## Abstract

**Simple Summary:**

Brain metastases arising from breast cancers, occur in about 20% of women with a poor-survival outcome. Unfortunately, most patients survive only up to eighteen months from diagnosis. Therefore, there is an urgent need to understand how these cancers survive in the brain. It is thought that the immune cells in the brain, together with brain resident cells, may provide a favorable environment for cancer growth. However, this is not very well understood at this point. We aimed to profile the cells found in these tumors, focusing on five different cell types based on the marker expressed by them. Our results indicate that certain molecules contained within the cancer and the surrounding environment are associated with poor survival. This suggests that these molecules might be important in brain metastasis. This finding is a step towards our understanding of how some patients with brain metastasis survive longer than others.

**Abstract:**

The heterogeneity of tumor infiltrating lymphocytes (TILs) is not well characterized in brain metastasis. To address this, we performed a targeted analysis of immune-cell subsets in brain metastasis tissues to test immunosuppressive routes involved in brain metastasis. We performed multiplex immunofluorescence (mIF), using commercially available validated antibodies on formalin-fixed paraffin embedded whole sections. We quantitated the subsets of immune-cells utilizing a targeted panel of proteins including PanCK, CD8, CD4, VISTA and IBA-1, and analyzed an average of 15,000 cells per sample. Classifying tumors as either high (>30%) or low (<30%) TILs, we found that increased TILs density correlated with survival. Phenotyping these TILs we found tumors with low TILs had significantly higher expression of the immune-checkpoint molecule VISTA in tumor cells (*p* < 0.01) as well as in their microenvironment (*p* < 0.001). Contrastingly, the tumors with high TILs displayed higher levels of microglia, as measured by IBA-1 expression. Low TILs-tumors displayed CD8+ T-cells that co-express VISTA (*p* < 0.01) significantly more compared to high TILs group, where CD8+cells significantly co-express IBA-11 (*p* < 0.05). These results were supported by RNA analysis of a publicly available, independent cohort. Our work contributes to a growing understanding of the immune surveillance escape routes active in brain metastasis.

## 1. Introduction

Metastatic tumors to the brain, which originate from extracranial primaries such as melanoma, breast and lung remain a significant clinical challenge. Life expectancy of patients after being diagnosed with brain metastases (BrM) can be measured in months [1] and BrMs make up a significantly higher proportion of adult brain tumors compared to primary brain tumors. Currently the gold standard for treating BrMs is surgery, chemotherapy, and radiotherapy. The advent of immunotherapies and their proven efficacy in a subset of BrM patients [2,3,4] has provided new hope for further therapeutic intervention. However, treatment alternatives still remain limited due to the lack of our comprehensive understanding of the heterogeneity of the microenvironment of the brain. This brain tumor microenvironment (TME) comprises various cell types that can regulate progression of the cancer and also response to therapy [5]. Given the unique adaptations of the brain TME [6,7,8,9,10,11], as well as the presence of uncommon cell types; microglia (MG), neurons, and astrocytes; and, the blood–brain barrier, makes it compelling to further address the critical question of how TME heterogeneity affects therapeutic efficacy.

Recent work has compared the immune landscape of BrMs and gliomas, where distinctive tumor specific features were discovered [12,13]. These ground-breaking studies observed more prominent infiltration of lymphocytes and leukocytes in BrMs compared to gliomas. However, targeted interrogation of the subtypes may be helpful in delineating prognostic cell subsets and specific phenotypes of these infiltrating lymphocytes [14]. In light of this biological question, we explored the infiltrating lymphocyte phenotypes of BrMs arising from breast cancers. We undertook a targeted approach of investigating five markers of interest: Pan cytokeratin (PanCK), CD4, CD8, V-domain immunoglobulin suppressor of T-cell activation (VISTA) and IBA-1 to elucidate the immune cell subtypes of BrMs that stratify according to the density of the tumor infiltrating lymphocytes (TILs). We utilized multiplex immunofluorescence (mIF) to address a number of questions in whole BrM sections. Do tumors with low density of TILs display a different phenotype compared to tumor with high density of TILs? Do the T-cell subtypes display any exhausted phenotypes? Does the density of the microglia differ in these tumors? We focused on the expression of the T-cell inhibitor molecule VISTA for our study as it is largely unexplored in BrMs from breast cancers.

VISTA was first identified by Wang and colleagues [15], where they discovered VISTA to have homology to the extracellular domain of B7 ligand programmed death ligand 1 (PD-L1). They found VISTA’s expression to be highly regulated on the myeloid antigen presenting cells and this inhibited T-cell proliferation and cytokine production. Since this discovery, knowledge on the role and function of VISTA in many cancers have been uncovered [16,17,18,19,20]. Furthermore, recent reports on mouse models of brain metastasis suggest VISTA blockade along with anti-PD L1 reduces BrM outgrowth and additionally, the release of cytokine Cxcl10 from tumor cells results in recruitment of VISTA expressing myeloid cells leading to T-cell suppression [21].

We interrogated the tumor-intrinsic as well as immune microenvironment specific expression of our marker panel. By exploring a targeted panel of markers within this selected cohort, we discovered that patients with reduced TILs density have increased VISTA+ immune cells, as well as tumor cells. This finding enhances our understanding of the immune-suppressive phenotypes of BrM patients with high and low density of TILs. Our study provides a clinical perspective in understanding the association of VISTA expression on the tumor as well as the microenvironment compartment of BrMs using whole tissue sections. This, in turn, may help us uncover new promising, immunotherapies, as well as answer fundamental biological questions related to BrM’s immunosuppressive TME.

## 2. Materials and Methods

### 2.1. Ethics

Ethical approval from the Human Research Ethics Committees of the Royal Brisbane and Women’s Hospital (RBWH; 2005000785) and The University of Queensland (HREC/2005/022) were obtained prior to the commencement of this study and de-identified samples were used for all the analyses performed. Treatment data were not available for all patients.

We compiled a cohort of brain metastases arising from breast cancers undergoing resection at the Royal Brisbane and Women’s hospital, Brisbane, Australia (*n* = 36). Based on the amount of necrosis and tissue availability, we performed multiplex immunofluorescence (mIF) on 20 whole tissue sections from 20 patients.

### 2.2. TILs Scoring

Immune cell infiltration was scored on hematoxylin and eosin-stained whole sections, within the boundary of the tumor by a pathologist (WGK). As there are no set diagnostic criteria for scoring TILs in brain metastases, for our research purposes we adapted the guidelines for TILs assessment from the “International Immuno-Oncology Biomarker Working Group” [22]. Immune infiltrate was quantitated by the area occupied by mononuclear inflammatory cells (lymphocytes and plasma cells) over total stromal area. TILs within the borders, invasive edges and stroma of the metastatic brain tumors were included in the evaluation. The proportion of TILs within the stromal area was measured in percentage and initially, criteria was set as per the guidelines. However, we re-stratified this criterion to > or <30% TILs, as this showed the best stratification within our cohort.

### 2.3. Transient Knockdown

MDA-MB-468 cells were purchased from the American Type Culture Collection (Manassas, VA, USA) and grown under standard culture conditions. Transient knockdowns were performed with mycoplasma free cells using 100 nM siRNAs comprising of three different probes (Gene Pharma, Shanghai, China) and FugeneHD (Promega, Madison, WI, USA) for 24 h. The cells were fixed in 10% neutral buffered formalin (Sigma-Aldrich, St Louis, MO, USA) after 24 h and embedded in paraffin for antibody validation by immunohistochemical analysis.
sense (5′-3′)antisense (5′-3′)CCGUAUUCCCUGUAUGUCUTTAGACAUACAGGGAAUACGGTGCAAAGAUGCACCAUCCAATTUUGGAUGGUGCAUCUUUGCTTGCAACAUUCAAGGGAUUGATTUCAAUCCCUUGAAUGUUGCTT

### 2.4. mIF and IHC

Four-micron thick sections were used to perform mIF for detecting CD4, CD8, VISTA, PanCK and IBA-1 using Tyramide Signal Amplification (TSA) (Opal Kit, Perkin Elmer, Waltham, MA, USA) according to the manufacturer’s instructions to detect. The sections were processed, and the images were acquired using the Vectra 3.0 Automated Quantitative Pathology Imaging System and analyzed using InForm software (v2.4.1, Perkin Elmer, Waltham, MA, USA). An average of 11 ROIs per tissue section were utilized to perform the analysis. As the analysis of the study is based on using algorithms to differentiate between the markers, we performed a thorough initial optimization to avoid false double positives. This was at first achieved by optimizing the dilution of the antibodies at different concentrations and on different channels. We found that VISTA (1:500, Opal 520; HPA007968, Sigma, Missouri, USA), CD4 (1:1000, Opal 570; M7310, Dako, Glostrup, Denmark), PanCK (1:200, Opal 620; AE1-AE3-M3515, Dako, Glostrup, Denmark), IBA-1 (1:50000, Opal 650; EPR16588, Ab178846, Abcam, Cambridge, UK), CD8 (1:1500, Opal 690; C8-114B, M7103, Dako, Glostrup, Denmark) gave us the best distinction with no bleed over of the channels. We also trained the InForm software to avoid nuclei, which could be colocalizing, hence, ensuring to circumvent any false double positives. Furthermore, using sections of only 4-µm in thickness aided us in bypassing colocalized nuclei, as one nucleus is usually about 5–6 µm.

IHC was also performed on 4-µm whole sections using the MACH1 Universal HRP-Polymer Detection Kit (BioCare Medical, Pacheco, CA, USA) according to the manufacturer’s instructions. Briefly, the sections were dewaxed, de-paraffinized and rehydrated in decreasing concentrations of alcohol (100–70%). Heat-induced antigen retrieval was performed using sodium citrate buffer (0.01 M, pH 6.0) at 110 °C for 10 min in a decloaking chamber (BioCare Medical). The sections were treated with 0.3% hydrogen peroxide for 30 min to remove endogenous peroxidases and then with MACH1 sniper blocking reagent (BioCare Medical) to avoid non-specific antibody staining. Primary antibody diluted in DaVinci Green Diluent (BioCare Medical) was applied to the sections and the slides were incubated for 3.5 h at RT in a humidified slide chamber. MACH-1 anti-rabbit secondary antibody conjugated to horseradish peroxidase was applied for 30 min at RT, followed by the diaminobenzidine (DAB) chromogen substrate for 5 min. The slides were counterstained with Hematoxylin for 4 min and cover-slipped using DPX mountant (Sigma Aldrich, St Louis, MO, USA). For analysis, the slides were scanned using an Aperio AT Turbo (Leica Biosysems, Wetzlar, Germany) at 40× magnification. For multiplex immunofluorescence and cell subset analysis, we used Vectra Polaris (Akoya Biosciences, Marlborough, MA, USA) and FCS express image cytometry software (De Novo Software, Pasadena, CA, USA).

### 2.5. Dataset Analysis

Raw gene counts were initially filtered, removing any gene with less than 1 count per million in at least 2 samples. The raw data were subsequently normalized using the trimmed mean of M values (TMM) in the edgeR package, resulting in an expression matrix with 226 samples and 18,735 genes. To rank the expression of VISTA, genes within each sample were sorted according to their expression, with the genes having the highest expression receiving the highest rank and vice versa for the gene with the lowest expression. To perform the ranking, the rank function in base R was used using default parameters. For investigating the gene counts from the RNAseq results, raw counts were downloaded from https://joycelab.shinyapps.io/braintime/ (accessed on 5 January 2021) and Log_2_ transformed and plotted in Prism software.

### 2.6. Statistical Analysis

All statistical analysis and the preparation of graphs were achieved by using GraphPad Prism software (v8.2). The data were analyzed using non-parametric *t*-tests with *p*-values < 0.05 considered to be significant.

## 3. Results

### 3.1. Cohort Description and Image Cytometry

**Figure 1:**
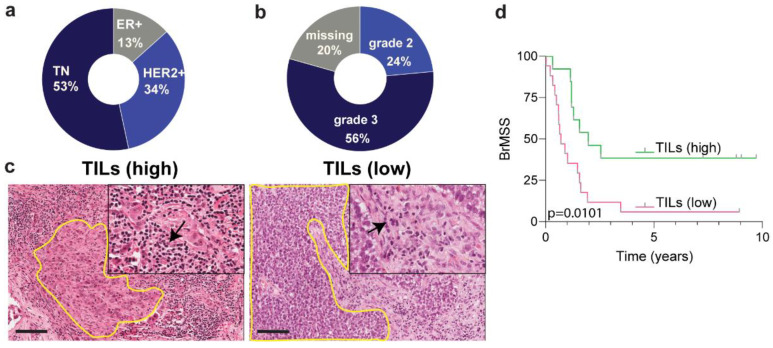
Cohort demographics and survival analyses. (**a**) Subtype breakdown of the primary breast cancers giving rise to the brain metastases; (**b**) tumor grade breakdown of the cohort; (**c**) hematoxylin and eosin stained representative image for TILs scoring and grouping; (**d**) Kaplan–Meier curve analysis showing brain metastasis specific survival of the high TILs and low TILs groups; TILs are indicated by arrows and yellow demarcated lines show the tumor. Scale bar 100 µm.

In this selected cohort, the median age at which the primary breast cancer was diagnosed was 45 years, with brain metastasis diagnosis at 47 years; median time to develop brain metastasis was 25 months from primary diagnosis. A majority of the 36 patients within this study (53%) had triple-negative BC-(TN) followed by HER2+ at 34%, and ER+ at 13% (Figure 1a). These breast cancers were also high grade with 56% patients falling into the grade 3 category and 24% into grade 2 (Figure 1b). The tumor infiltrating lymphocytes were scored according to the international TIL working group [22], and we restratified the groups into TIL density criteria based on >30%, high or <30%, low (Figure 1c). This segregation was significantly prognostic (*p* < 0.01) for brain metastasis specific survival (BrMSS) with a hazard ratio of 2.9 (Figure 1d).

**Figure 2:**
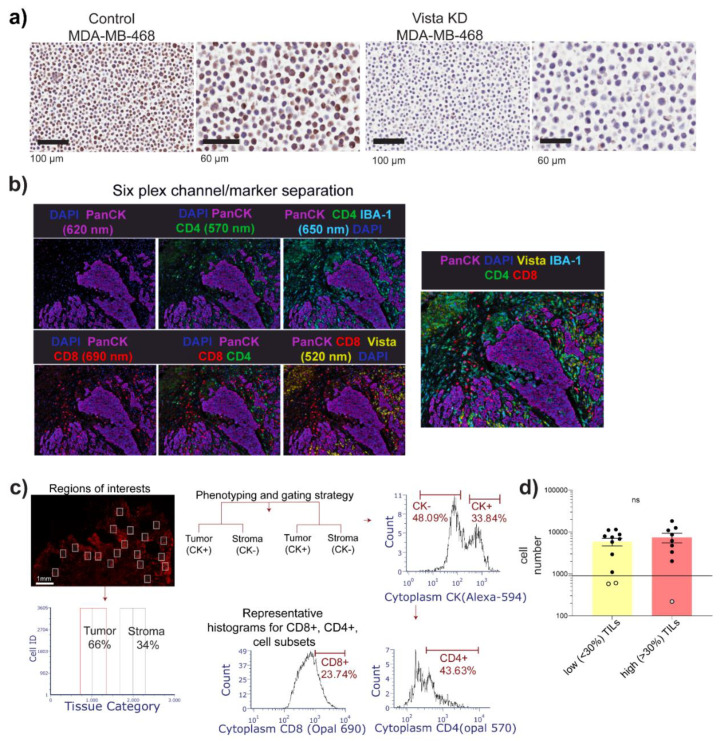
Imaging, tissue, and cell segmentation. (**a**) VISTA antibody validation on MDA MB 468 cells using IHC on scrambled control and siRNA against *VSIR* gene. (**b**) Spectral unmixing and separation of the fluorochromes highlighting the different cell subsets; (**c**) data integration and representative gating strategy for single-cell subset analysis in different regions of the samples; example of the ROIs in a case followed by separation of the tumor and stroma regions based on PanCK expression. These parent cell populations were further gated for CD8+, CD4+ cell subsets. (**d**) Average cell numbers from each group being analyzed in the study; each dot represents a case; ns = non-significant.

We performed transient knockdown of the *VSIR* gene on the breast cancer cell line MDA-MB-468 to test the specificity of the VISTA antibody. Antibodies against the other markers were extensively used and well characterized, eliminating the need to re-validate them. As demonstrated in Figure 2a, at high and low magnifications we observe positive staining for the VISTA protein in cells that received scrambled control sequence. Contrastingly, in the cells that received the short interfering RNA sequence against *VSIR* we observe no staining at the protein level. After validation of the antibody, we performed multiplex immunofluorescence (mIF) labeling using tyramide signal amplification (TSA) to profile CD4, CD8, VISTA, Pan cytokeratin (PanCK) and IBA-1 positive cell subsets within this cohort. As mentioned previously, based on necrosis and tissue availability, we were able to perform mIF on 20 samples. Images were acquired on the Vectra automated quantitative digital pathology platform and data were processed using InForm software’s standardized workflow tool. Spectral unmixing precisely separated the staining patterns of each protein by employing the distinctive features of the dyes and was performed to ensure no bleed through or overlapping of adjacent fluorochromes (Figure 2b), thus limiting false positive results. Representative images of the separation of DAPI, PanCK at 620 nm, CD4 at 570 nm, IBA-1 at 650 nm, CD8 at 690 nm, and VISTA at 520 nm, are shown in Figure 2b; a clear distinction of the cellular subsets is observed at the different wavelength channels for each marker. Following tissue and cell segmentation, we integrated the respective segmentation dataset employing FCS express 6 cytometry software. This enabled us to analyze the multiplexed images from 10–15 randomly selected regions of interest for each case (Figure 2c). We quantified the multi-parameter spatial (different regions of the tumor), as well as single-cell cluster phenotypes within our dataset. The data were first scrutinized at the tissue level with tumor and tumor-associated area/stroma separated on a histogram (Figure 2c). We also visualized the data on histograms for each tissue region to separate the CK+ (tumor/epithelial cells) and CK− (non-tumor cells), followed by CD8+ and CD4+ subsets (Figure 2c) and approximately 15,000 cells per case were analyzed. It should be noted that, for immunofluorescence data based on intensity, a small population of cells overlap between two markers as can be seen in the histograms (Figure 2c), therefore, these population of overlapping cells were not included in the analysis. Three samples had a cell count of <1000 (Figure 2d) and as the cell count was too low with respect to whole sections, these samples were excluded from further analysis. Hence, we analyzed 9 samples from low TILs group and 7 from high TILs group (Figure 2d).

### 3.2. Increased VISTA and IBA-1 Expression in the TME

**Figure 3:**
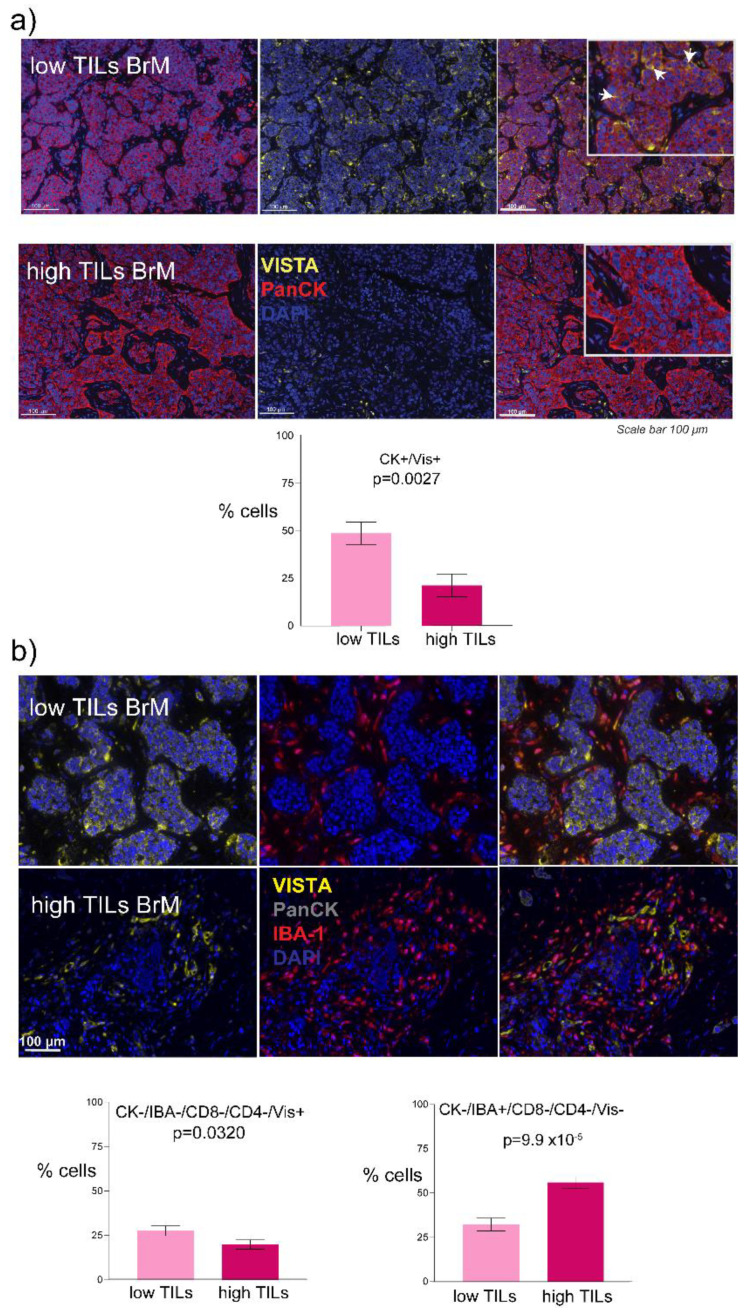
VISTA and Iba1+ cellular subtypes. (**a**) VISTA expression on the tumor cell surface of the low vs. high TIL groups and the associated bar graph; arrows indicate vista positive tumor cells. (**b**) Images represent the expression of VISTA and IBA1+ cells and bar graphs show VISTA and IBA1+ exclusive cells between the low and high TIL groups, respectively.

We segmented the tissue regions into tumor and stroma and analyzed each compartment to delineate cellular phenotypes. We considered cells showing positive expression of PanCK to be tumor epithelial cells and quantified the subset of TILs as indicated in Figure 2c. Initially, we considered single marker positive cell subsets and found that VISTA was expressed on the tumor cell surface in all samples however, the proportion of VISTA+ tumor cells differed between the groups (Figure 3a). The percentage of tumor cells that expressed VISTA on their surface was 50% in the low TIL group compared to 23% in the high TIL group (*p* = 0.0027; Figure 3a).

We also found its exclusive expression in 27% of the low TIL group, which was significantly higher than 19% in the high TIL group (*p* = 0.0320; Figure 3b). Interestingly, VISTA has been reported to be highly expressed by microglia and especially differentially expressed in central nervous system (CNS) pathologies [23]. Therefore, we next asked if there was an association between VISTA and the microglial marker IBA-1 expression in the TME. VISTA and IBA-1 co-expression were found to be present in about 25% of the microglia (Supplementary Figure S1i). Interestingly, 40% of both groups displayed negativity for all the markers used in the study (Supplementary Figure S1ii). As the presence of microglia in the brain TME has been previously described [24] so we investigated if there was a difference in the proportion of microglial cells between low and high TILs groups. We observed that 60% of the TME cells were Iba1+ in the high TILs group (*p* = 9.9 × 10^−6^; Figure 3b) compared to 30% in the low TIL group.

### 3.3. Phenotypes of the CD8+ T-Cells

**Figure 4:**
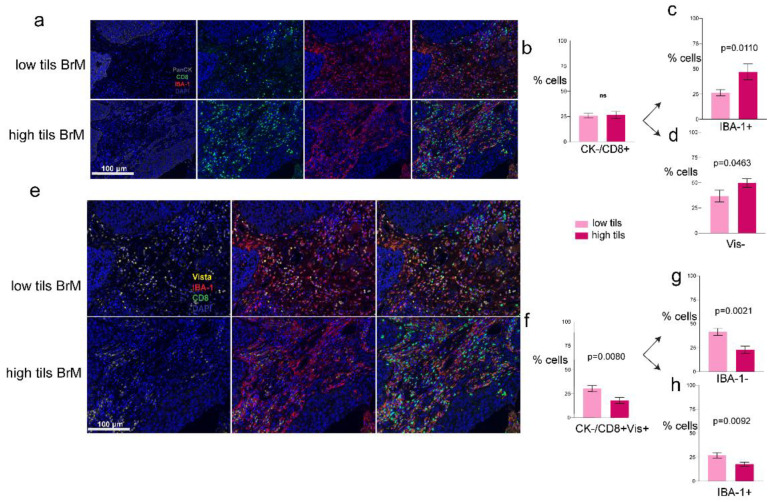
CD8+ T cell populations with arrows indicating the direction of the subsequent gated cell populations within the parent population. (**a**) Representative images of mIF: CK, CD8, and IBA-1; (**b**) CD8+ gated population of cells comparison between the low and high TIL groups; (**c**) bar graphs showing IBA-1+ cells within the CK−/CD8+ parent populations; (**d**) VISTA negative cells within the CK−/CD8+/IBA-1+ cells; (**e**) representative images of mIF: CD8, VISTA and IBA-1; (**f**) CK−/CD8+ gated parent population with VISTA expression; (**g**) far graphs showing IBA-1- cells within the CK−/CD8+Vis+ parent populations; (**h**) IBA-1+ cells within the same parent populations.

We investigated the CD8+ and CD4+ T-cell phenotypes in our cohort. The CD4+ T-cells did not differ significantly in their proportions in low and high TIL groups, 30% of CK− cells were CD4+ in the low TILs and 38% in the high TILs group (Supplementary Figure S1iii). We then performed analysis on the subpopulations within the CD4+ subtypes (Supplementary Figure S1iv–viii). We found 37% and 40% of CD4+ cells co-expressed IBA-1 in low and high TILs, respectively (Supplementary Figure S1iv). Within CD4+ T cell population, subsets of IBA-1+/VISTA+ (Supplementary Figure S1v) and IBA-1+/VISTA- (Supplementary Figure S1vi) were found to be similar in terms of their proportions in both groups. Furthermore, analysis of CD4+ subsets with VISTA+ (Supplementary Figure S1vii) and VISTA+/IBA-1- (Supplementary Figure S1viii) were also found to be similar in the two groups. In tumors with high and low TILs (Figure 4a), 25% and 26% of CK− cells were CD8+ T-cells, respectively (Figure 4b). Interestingly, this changed for double positive cells; where 46% of CD8+/IBA-1+ were found to be present in the high TILs groups compared to 26% in the low TILs (*p* = 0.0110; Figure 4c). We investigated if this was true when triple population subsets were analyzed, and this pattern remained the same, with the high TIL group displaying higher (49%) CD8+/IBA-1+/VISTA- populations (*p* = 0.0463; Figure 4d). When we examined VISTA expression within CD8+ populations (Figure 4e) we found 30% of CD8+ T-cells expressed VISTA in the low TIL group compared to 18% in the high TIL group (*p* = 0.0080; Figure 4f). Intriguingly, when we investigated IBA-1 positivity within the CD8+/VISTA+ population, we found a similar trend with CD8+/Vis+/IBA-1+ or IBA-1- cells; where low TIL groups had higher amounts of both populations, 44% (*p* = 0.0021; Figure 4g) and 26% (*p* = 0.0092; Figure 4h) compared to high TIL tumors, respectively.

### 3.4. Validation of mIF Using IHC and RNA Expression Analysis

**Figure 5:**
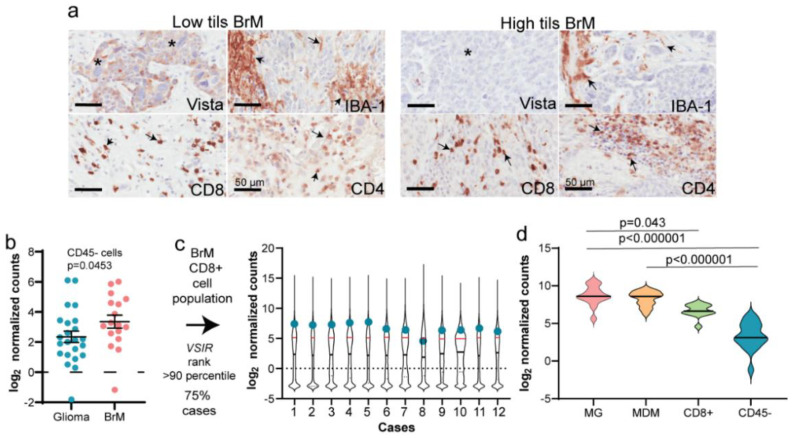
VISTA expression analysis using single IHC, as well as RNA analysis, in the Klemm dataset. (**a**) Representative images of standard IHC staining on BrMs sections with * representing tumor cells and arrows indicating positive staining. (**b**) *VSIR* gene expression in BrMs and gliomas within CD45- population in the Klemm RNAseq dataset [12]; (**c**) ranking of VSIR in CD8+ population within BrM cases; (**d**) within BrMs expression levels of VSIR in Microglial (MG), monocyte-derived macrophages (MDMs), CD8+ and CD45- populations.

After quantifying the phenotype of the TILs using mIF, we next attempted to validate our findings using standard immunohistochemistry of VISTA, IBA-1, CD4 and CD8 on eight randomly selected whole sections from the same samples where we had performed mIF; four from low TILs and four from high TILs. Upon semi-quantitative scoring, VISTA was positively stained on the tumor cells for three out of four cases in the low TIL group. In the high TIL tumors, no tumor cell positivity was evident on single IHC for VISTA (Figure 5a). IBA-1 (Figure 5a) was also found to be highly expressed in the TME of high TIL BrMs compared to low TILs (Figure 5a). We also observed a similar pattern of positive staining for CD8 and CD4 in the TME in low and high TIL tumors with no evident difference (Figure 5a). To further delineate the expression of VISTA in BrMs, we explored the Klemm dataset [12], where after flow cytometry assisted separation of immune cell and non-immune cells, they performed RNA-sequencing on BrMs and glioma samples. We examined the expression of *VSIR* in BrMs and gliomas in the non-immune (CD45-) population as a comparison between primary and metastatic brain tumors as the Klemm study highlighted that there are differences between these tumors. We found BrM non-immune cells to have a significantly higher level of VISTA expression (*p* = 0.0453; Figure 5b). Ranking of VSIR gene within the CD8+ population in BrMs revealed that in 75% of cases, VISTA’s abundance is in the top 10th percentile (top quartile) of all ~18,000 genes expressed in this cell subtype (Figure 5c). We also compared the expression of *VSIR* in the microglial (MG), monocyte-derived macrophages (MDMs) and non-immune population (CD45-) within BrMs. Interestingly, we found that in BrMs *VSIR* expression was significantly higher in the cells from myeloid lineage, especially microglia (Figure 5d). This population had significantly higher expression of *VSIR* compared to CD8+ (*p* = 0.043; Figure 5d), as well as CD45- cells (*p* < 0.000001; Figure 5d). The expression levels were comparable between the MDMs and CD8+ cells, however, *VSIR* expression was higher in MDMs compared to CD45- cell (*p* < 0.000001; Figure 5d).

## 4. Discussion

Brain metastasis from the breast is one of the common causes of mortality in women, and the poor quality of life and the associated morbidity makes it compelling to understand the disease better. An emerging and evolving concept within the brain TME is the existence of the complex landscape of cells within this unique organ. The positive brain tumor responses to immune checkpoint inhibition raises optimism that extensive understanding of the biology of the tumor will lead to better management. There is hope that developing a comprehensive understanding of the TME will lead to design of more effective immunotherapies. In spite of emerging data associating tumor infiltrating lymphocytes to prognosis in BrMs [25,26,27], the immune cell subsets and phenotypes within BrMs are not well-defined. We analyzed a targeted panel of immune-related proteins; CD4, CD8, IBA-1, and VISTA which have not yet been described in brain metastasis in either single- or co-expression context by employing a mIF approach. We interrogated how the TILs in patients with a better prognosis differ in their phenotype, especially CD8+ and CD4+ T-cells, compared to the ones with poor prognosis. Our study has shown a novel immune checkpoint, VISTA, to be highly expressed in brain metastasis and its expression is higher in the group of patients who have a worse prognosis compared to the high TILs group. We have also provided insights into the VISTA co-expressing immune cell subsets within our selected cohort. We found IBA-1+ cells to be more pronounced in BrMs with high density of TILs and we also observed CD8+ IBA-1+ cells.

Our data showed that VISTA is highly expressed on the tumor cell surface of BrMs and this was significantly higher in the group with low density of TILs. Overall, we observed an enhanced expression of VISTA in the tumor compartment as well as in the microenvironment of patients with low density of TILs. Interestingly these patients also had a poorer prognosis compared to the high TILs group. In this setting, VISTA might be manifesting immune-modulatory effects, especially in tumors with a low density of TILs. However, we cannot eliminate the possibility that VISTA expression on the tumor cell surface may have caused less TILs to infiltrate the area. VISTA is a negative immune checkpoint regulator highly expressed in myeloid cells and its expression is known to be suppressive on resting as well as activated CD4+ and CD8+ T-cells [28]. Exclusive VISTA expression on the CK negative or TME compartment is interesting but perplexing as we cannot rule out if these are CD3+ T-cells or a myeloid population. Further phenotyping is required with specific T-cell and myeloid cell markers to pinpoint this unique population. Our findings on VISTA being expressed by epithelial cells confirms data in other cancers [20,28,29,30,31,32]. In non-small cell lung carcinomas (NSCLC), VISTA was found to be expressed in a majority of NSCLCs and, contrastingly, this expression associated with lymphocyte infiltration and PD-L1 expression [29]. In ovarian cancer, expression of VISTA has contradicting reports. On the one hand, two studies indicated VISTA to be highly enriched in tumors leading to a poor prognosis and advanced disease while on the other, one study shows VISTA to be associated with good prognosis. Mulati and colleagues found VISTA to be expressed in 91% of the samples and in an animal model bearing ovarian tumors, anti-VISTA antibody increased the longevity of the treated animals by reducing the tumor burden. In vitro studies showed that tumor cell expression of VISTA caused immune evasion by suppressing T-cell proliferation and cytokine production [28]. Additionally, VISTA expression has been linked to the progression of the disease in ovarian cancers. The authors noted that expression of VISTA increased with advanced disease and lymph node metastasis [20]. Contrastingly, VISTA’s expression has also been shown to associate with a favorable prognosis in a different cohort of high-grade ovarian cancers. This retrospective study had used tissue microarrays to assess the expression of VISTA on tumor cells, as well as in immune cells, therefore, intra-tumoral heterogeneity would not be accounted for [32]. These two studies had different scoring criteria, and the study by Liao and colleagues used whole sections which might have provided a higher accountability for the heterogeneity within the section. Nevertheless, more in-depth analysis is required to understand the contrasting associations of VISTA in ovarian cancer. Our observation of co-expression of VISTA on CD8+ T cells on low TILs density can also be extrapolated to suggest that perhaps VISTA expression is causing immune evasion.

With respect to Central Nervous System metastatic disease, recent work by Guldner and coworkers have identified brain resident myeloid cells to be inducing an immunosuppressive microenvironment, thus promoting metastasis [21]. This study generated mouse models of BrMs to derive functional understanding of the myeloid cell population in this disease. When they performed mechanistic in-vitro and in-vivo studies they found C-X-C motif chemokine ligand 10 (Cxcl10) promoted BrMs, and they further characterized to elucidate the mechanism behind this. Investigation of publicly available human CNS myeloid RNAseq datasets, they found increased expression of Vsir (VISTA) and CD274 (PD-L1) in the myeloid cells [21]. In addition, they found co-inhibition of PD-L1 and VISTA in a mouse model of brain metastasis reduced the metastatic burden. Our results are complimentary to their finding where we have shown increased VISTA in a sub-population of patients with BrMs and its expression is an indicator of poor prognosis. Furthermore, we observe an enhanced expression of VISTA in BrMs compared to gliomas as well as myeloid lineage cells have more pronounced VISTA in our mIF study and in the Klemm dataset [12]. To our knowledge our study is the first to show this differential expression of VISTA in the tumor as well as in CD8+ T-cells in a BrM clinical cohort.

Brain resident macrophages/microglia perform numerous roles in brain health and disease [33]. With respect to BrMs, microglia are known to be multifaceted in their function such as promoting proliferation, angiogenesis and invasion in BrMs [34]. Recent work by Simon and co-workers, highlighted using intra-vital microscopy how microglia are recruited and activated after xenotransplantation of breast cancer cells into the brain [35]. This study showed that microglial activation and accumulation around the lesion can cause infiltration of immune cells as well as aberrant electrical activity in the brain. Similarly, in a mouse model of BrM, microglial accumulation has been associated with increased tumor burden. When selective depletion of the anti-inflammatory microglial phenotype was carried out, a reduction in tumor burden was observed [36]. These studies highlight the importance or microglia in brain metastasis. For this study, we used a common microglial marker IBA-1 in our panel to investigate the expression levels and associations of IBA-1+ microglia.

Our findings suggest increased microglia in the tumors with high density of TILs and we also observe increased CD8+ T-cells that co-express IBA-1. Simultaneous accumulation of microglia and infiltration of immune cells has been shown in a mouse model of BrM [35]. Although no mechanism is known for this association, this accumulation was found to reduce the density of tumor cells in the brain [35]. We observed increased microglia in the high TILs group which also had a better prognosis. This suggests that perhaps increased microglia may have caused anti-tumor immunity. Additionally, our perplexing finding of double positive CD8+/IBA-1+ cells, leads us to suggest that these double positive cells may be representing pro-inflammatory M1 macrophages. This is plausible because Boddaert and colleagues used a rat model for stroke to demonstrate increased CD8 expression in activated macrophages using IBA-1 as a marker. Their results corroborate our finding of double-positive macrophages. Furthermore, in-vitro analysis revealed CD8 stimulation caused repolarization of M2 to M1 phenotype along with highly proliferative IBA-1+CD8+ cells [37]. If this is true for our findings, then this co-expression could play a protective role in BrMs. It is known that microglia can exist in two states, anti-inflammatory which facilitates invasion, angiogenesis and tumor growth [34,38]; and pro-inflammatory which can release proinflammatory cytokines, eliciting a T-cell response [39]. Pro-inflammatory microglial phenotype switch can infer a microglial population which may have caused anti-tumor immune response [40]. Nonetheless, we cannot rule out the possibility of false double positive cells; however, we strongly believe this is highly unlikely. Future studies are required to investigate a larger population of resected tumors using flow cytometry to see if double positive CD8+/IBA-1+ are a consistent phenotype in BrM.

We appreciate the limitations of this study such as the size of the cohort, which is modest, though it should be noted that assembling BrM cohorts that include clinical follow-up information is challenging, particularly given an increasing trend for breast cancer patients to have targeted radiotherapy of small, early lesions rather than surgery. We have used commercially validated antibodies however, batch variability might still play a role and therefore, future studies should focus on employing various commercially available antibodies to test the suggested phenotypes of TILs in BrM from this body of work. In addition, we cannot eliminate the effect of treatment that may be impacting the TILs phenotype as our patient population ranges from early to late 2000s and the treatment regimen has drastically changed within the last decade. For example, the emerging use of Pembrolizumab (anti-PD-L1) in the metastatic triple-negative breast cancer setting. It would be crucial to test VISTA expression in a patient population treated with current immunotherapy regimens as that might provide novel insights into treatment response. Furthermore, BrMs arising from different primaries have different natural history and tumor architecture which needs to be further elucidated. Finally, future functional studies delineating the mechanisms of differential expression of VISTA and microglia in BrMs are warranted.

Overall, we utilized mIF as a tool to provide a proof-of-concept study to demonstrate TME-distinctive changes in the TILs of BrM. Through this, we have defined the expression of a new immune checkpoint molecule in a clinical cohort of BrMs.

## 5. Conclusions

Brain metastasis from the breast is one of the common causes of mortality in women, the poor quality of life and the associated morbidity makes it compelling to understand the disease better. An emerging and evolving concept within the brain TME is the existence of the complex landscape of cells within this unique organ. The positive responses to immune checkpoint inhibition in brain tumors raises optimism that extensive understanding of the biology of the tumor will lead to better management. There is hope that developing a comprehensive understanding of the TME will lead to design of more effective immunotherapies. Our study found that density of infiltrating lymphocytes stratifies patients with brain metastasis; specifically, BrMs with low TILs have a poorer prognosis compared to high TILs group. Within each of these two groups we defined two different phenotypes, A and B (see graphical abstract). We found a novel immune checkpoint, VISTA, to be highly expressed in brain metastasis and its expression is higher in the group of patients who have fewer infiltrating lymphocytes to the high TILs group. Within this selected cohort of brain metastasis samples, we have also provided novel insights into the VISTA co-expressing immune cell subsets. We found IBA-1 expressing cells to be more pronounced in BrMs with high density of TILs and we also observed a unique population of CD8 + IBA-1 cells. Further studies are required to elucidate the mechanisms of differential expression of VISTA and microglia in BrMs.

## Supplementary Materials

The following are available online at https://www.mdpi.com/2079-7737/10/5/425/s1, Figure S1: Quantified bar graphs of other cytokeratin negative and CD4+ subsets within the groups.
