# Supplementary figures and images for: Characterization of Immune Cell Subsets of Tumor Infiltrating Lymphocytes in Brain Metastases
Source: Biology (Basel). 2021 May 11;10(5):425. doi: 10.3390/biology10050425 (PMC8150725; doi:10.3390/biology10050425)

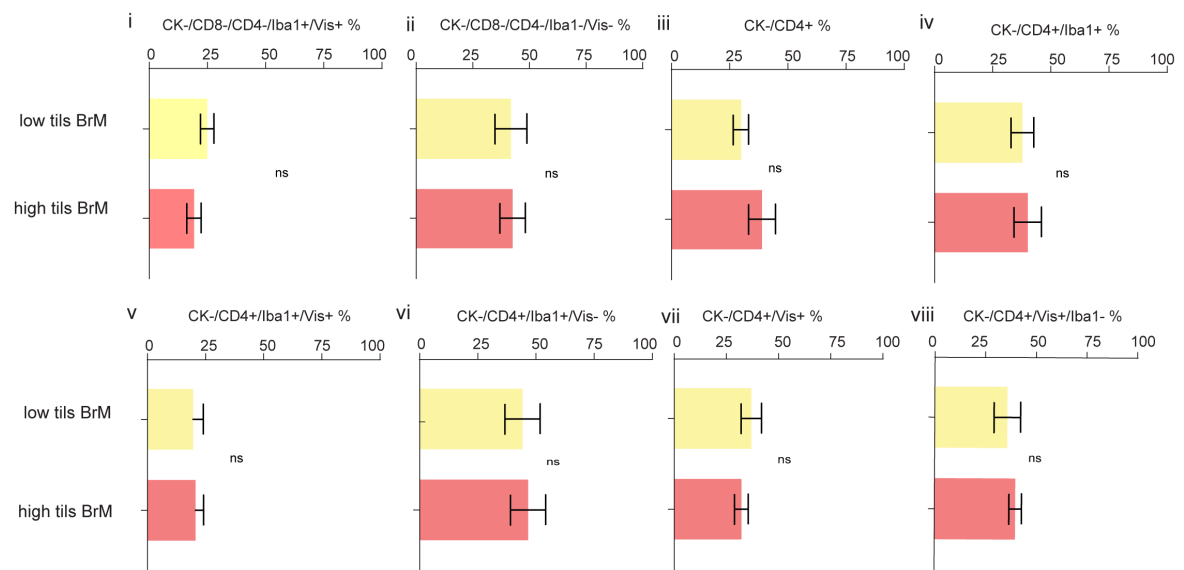

**Figure S1.** Quantified bar graphs of other cytokeratin negative and CD4+ subsets within the groups.

Supplement: Supplementary file 1 [file biology-10-00425-s001.zip › biology-1144699-supplementary.pdf]
